# Using Big Data to Assess Prescribing Patterns in Greece: The Case of Chronic Obstructive Pulmonary Disease

**DOI:** 10.1371/journal.pone.0154960

**Published:** 2016-05-18

**Authors:** Kyriakos Souliotis, Chara Kani, Manto Papageorgiou, Dimitrios Lionis, Konstantinos Gourgoulianis

**Affiliations:** 1 Faculty of Social and Political Sciences, University of Peloponnese, Corinth, Greece; 2 Centre for Health Services Research, Medical School, University of Athens, Athens, Greece; 3 Medicines Division, National Organization for Healthcare Services Provision (EOPYY), Athens, Greece; 4 Department of Social and Educational Policy, University of Peloponnese, Corinth, Greece; 5 Department of Respiratory Medicine, Medical School. University of Thessaly, Larissa, Greece; University of Athens, Medical School, GREECE

## Abstract

**Introduction:**

Chronic Obstructive Pulmonary Disease (COPD) is one of the top leading causes of death and disability, and its management is focused on reducing risk factors, relieving symptoms, and preventing exacerbations. The study aim was to describe COPD prescribing patterns in Greece by using existing health administrative data for outpatients.

**Methods:**

This is a retrospective cross-sectional study based on prescriptions collected by the largest social insurance fund, during the first and last trimester of 2012. Selection criteria were the prescription of specific active substances and a COPD diagnosis. Extracted information included active substance, strength, pharmaceutical form and number of packages prescribed, diagnosis, time of dispensing, as well as insurees’ age, gender, percentage of co-payment and social security unique number. Statistical analysis included descriptive statistics and logistic regression.

**Results:**

174,357 patients received medicines for COPD during the study period. Patients were almost equally distributed between male and female, and age above 55 years was strongly correlated with COPD. Most patients received a long-acting beta agonist plus inhaled corticosteroid combination (LABA +ICS), followed by long-acting muscarinic agonist (LAMA). 63% patients belonging in the 35–54 age received LABA+ICS. LAMA was prescribed more frequently among males and was strongly correlated with COPD.

**Conclusion:**

The study provides big data analysis of Greek COPD prescribing patterns. It highlights the need for appropriate COPD classification in primary care illustrating the role of electronic prescribing in ensuring appropriate prescribing. Moreover, it indicates possible gender differences in treatment response or disease severity, and the impact of statutory co-payments on prescribing.

## Introduction

The World Health Organization has classified Chronic Obstructive Pulmonary Diseases (COPD) as the fifth leading cause of death in 2002 and estimates that in 2030, it will be the third leading cause of death worldwide.[1] The incidence of COPD is about 2–4% in the UK,[2] while its prevalence among adults varies from 8% to 10% in countries with regular measurements.[3]

COPD also exerts high impact on patients’ quality of life, as measured by Disability-Adjusted Life-Years (DALYs), and is associated with a significant social impact. Under this prism, a study of the World Bank for the total burden related to COPD estimates that COPD will be responsible for 4.1% of total DALYs and the fifth leading cause of DALYs lost worldwide by 2020.[4]

The economic burden of COPD is high,[5] while there is a definite relationship between the cost and the severity of the disease. The management of the disease includes pharmacologic treatment, oxygen therapy, hospitalization[6] and medical visits and is related to productivity reduction and premature death.[7] In Europe the total direct cost of pulmonary diseases corresponds to 6% of the total health budget, while COPD is responsible for 56% of this cost (38.6 billion).[8]

The standard of care of stable COPD includes the use of short-acting beta agonists (SABA), long-acting beta agonists (LABA), short-acting anticholinergics (SAMA), long-acting anticholinergics (LAMA), corticosteroids (ICS) which are administered by inhalation, theophylline syrup, and roflumilast tablets. A number of treatment guidelines regarding the use of the above-mentioned medicines according to the severity/ stage of the disease have been published by medical associations and scientific bodies worldwide.[9]

In Greece, in 2004, the prevalence of COPD in the population over 35 years was estimated at 8.4%, with a higher prevalence identified in men (11.6%) compared to women (4.8%).[10] However, in the last years, COPD prevalence in females appears to be on the rise as well, as a result of increased smoking among women.[11]

The management of COPD in the Greek health system is conducted at both inpatient and outpatient level, however, the first point of contact is usually a general practitioner, an internist or (less often) a respiratory medicine specialist. Outpatient physicians offer primary care services either at private practices contracted with the largest social security fund, i.e. the National Organization for Healthcare Services Provision (EOPYY), or at the health centers of the National Primary Care Network (PEDY).

In this context, the aim of the present study was to describe COPD prescribing patterns in Greece by using existing health administrative data for a large number of outpatients.

## Methods

This is a non-interventional, cross-sectional observational study of the pharmaceutical treatment in stable COPD among outpatients. Health administrative data were collected from the Central Unit of Prescriptions Processing (KMES) of EOPYY in Greece. KMES is a platform and business intelligence system which incorporates data from electronically prescribed and hand written prescriptions. It should be noted that e-prescribing penetration is above 95% in the Hellenic system for outpatients.

EOPYY is the largest social security fund (SSF) covering more than 90% of the insured population, and was created in 2012 from the merger of individual SSFs. In the first months of 2012 the four biggest SSFs (IKA, OAEE, OGA, OPAD) formed EOPYY while by November 2012 the majority of the remaining funds were also incorporated into EOPYY. EOPYY provides health insurance under a single benefits package for the Greek population, either through a network of contracted physicians, or through the health centers of PEDY (Primary National Health Network).

KMES is a structure that ensures recording, filing, processing and analyzing of all prescriptions submitted in the system by all the private pharmacies contracted with EOPYY. In the present study, KMES data on all medicines prescriptions dispensed by private pharmacies were retrieved for the time periods January to March 2012 and September to December 2012 (first and last trimester of 2012), according to the following criteria a) presence of a COPD diagnosis on the prescription b) prescription of specific therapeutic categories related to the treatment of COPD and active substances. The active substances included in the prescriptions extracted from KMES are presented in Table 1. It should be noted that the KMES database has the following characteristics: completeness of data (adequate individual patient’s designation by age, sex, unique security number and ICD-10 diagnosis code), consistency of data collection (longitudinal data) and ability to follow up individual patients.

**Table 1:** Active substances included in prescriptions extracted.

ATC	ACTIVE SUBSTANCE	THERAPEUTIC CATEGORY
**R01AD05**	BUDESONIDE	ICS
**R01AD08**	FLUTICASONE PROPIONATE	ICS
**R01AD12**	FLUTICASONE FUROATE	ICS
**R03AC02**	SALBUTAMOL SULFATE	SABA
**R03AC03**	TERBUTALINE SULFATE	SABA
**R03AC12**	SALMETEROL XINAFOATE	LABA
**R03AC13**	FORMOTEROL FUMARATE	LABA
**R03AC18**	INDACATEROL MALEATE	LABA
**R03AK04**	IPRATROPIUM BROMIDE MONOHYDRATE,SALBUTAMOL SULFATE	SABA+SAMA
**R03AK06**	FLUTICASONE PROPIONATE,SALMETEROL XINAFOATE	LAMA + ICS
**R03AK07**	BECLOMETASONE DIPROPIONATE,FORMOTEROL FUMARATE DIHYDRATE	LAMA + ICS
**R03AK07**	BUDESONIDE,FORMOTEROL FUMARATE DIHYDRATE	LAMA + ICS
**R03BA02**	BUDESONIDE	ICS
**R03BA05**	FLUTICASONE PROPIONATE	ICS
**R03BB01**	IPRATROPIUM BROMIDE	SAMA
**R03BB04**	TIOTROPIUM BROMIDE MONOHYDRATE	LAMA
**R03CC02**	SALBUTAMOL SULFATE	SABA
**R03DA04**	THEOPHYLLINE	Methylxanthine
**R03DX07**	ROFLUMILAST	Phosphodiesterase-4-inhibitors

SABA: Short Acting beta2-agonist, SAMA: Short Acting Muscarinic Antagonist, LABA: Long Acting beta2-agonist, LAMA: Long Acting Muscarinic Antagonist, ICS: Inhaled corticosteroid, PDE-4: Phosphodiesterase inhibitor.

ATC = Anatomical Therapeutic Chemical (ATC) Classification System.

The present study is a retrospective observational epidemiological study undertaken in a very large cohort conducted exclusively by examining health administrative data. Data regarding the active substance, strength, pharmaceutical form, time of dispensing, patient’s age and gender, diagnosis, number of packages, percentage of co-payment and patient’s unique social security number were extracted. The data received from the Health Reimbursement Fund for the purpose of this study where anonymized with respect to patients' identification. Permission for use of anonymized data was obtained by the administration of EOPYY (approval decision of the President / protocol number C31/906/22.06.2013), in accordance to the national legislation on the Protection of Individuals with regards to the Processing of Personal Data. The study has been approved by the Scientific Committee of the University of Peloponnese (No 0222).

Statistical analysis included descriptive statistics for epidemiological data and therapeutic categories, while logistic regression was used to assess possible correlation between patients’ characteristics (age, gender, quantity of medicines, co-payment percentage, therapeutic category-ATC4) and presence of COPD. For continuous variables the mean and standard deviation were calculated, while for categorical variables the number and the corresponding percentage were calculated. All performed analyses were done using the IBM SPSS Statistics 20 Software.

## Results

The total number of EOPYY’s insurees who received treatment for COPD during the study period was 174,357 with a mean age of 69.3±14.8 years. The descriptive statistics of the study population are presented in Table 2. In Greece a statutory patient co-payment of 0%, 10% and 25% on reimbursed price of each medicine is set. The percentage of the co-payment per disease category is set by a Ministerial Decree and is usually related to the severity and duration of a disease.

**Table 2:** Characteristics of studied sample.

Variable	Mean value+-SD / N (%)
Demographic characteristics	
Number of patients	174,357
Age(MEAN +- SD)	69.3± 14.8
Gender (Number of males (%))	90,645 (52%)
Prescriptions’ details	
Quantity prescribed	1.36± 0.9
Percentage of prescriptions with statutory participation 25% compared to 10%	112,752 (64.7%)
Type of prescribed medicines based on active substance	
LABA &_ICS (N (%))	92,755 (53.2%)
ICS (N (%))	60,177 (34.5%)
LAMA (N (%))	58,709 (33.7%)
LABA (N (%))	44,785 (25.7%)
SAMA (N (%))	15,987 (15.2%)
SABA_ & SAMA (N (%))	18,700 (10.7%)
Phosphodiesterase_4_inhibitors (N (%))	6,933 (4%)
Methylxanthine (N (%))	6,166 (3.5%)
SABA (N (%))	17,004 (2%)

SABA: Short Acting beta2-agonist, SAMA: Short Acting Muscarinic Antagonist, LABA: Long Acting beta2-agonist, LAMA: Long Acting Muscarinic Antagonist, ICS: Inhaled corticosteroid, PDE-4: Phosphodiesterase inhibitor.

The percentages of active substance use represent the number of insurees in the sample who actually received the medicine compared to the number of insurees who did not receive the active substance.

The distribution of study population according to age, gender and active substance groups is presented in Table 3.

**Table 3:** Distribution of COPD patients ((N, (%)) in age groups according to gender and active substance group treatment.

Age-group	Gender	ICS	SABA	LABA	SABA_SAMA	LABA_ICS	SAMA	LAMA	Methylxanthine	PDE-4
35–54	F	3747	1316	2339	396	7280	524	2115	163	189
		(32.9%)	(11.5%)	(20.5%)	(3.5%)	(63.9%)	(4.6%)	(18.6%)	(1.4%)	(1.7%)
	M	2409	823	1943	330	5169	370	2370	159	221
		(29%)	(9.9%)	(23.4%)	(4%)	(62.1%)	(4.4%)	(28.5%)	(1.9%)	(2.7%)
55–74	F	6156	3400	9008	2492	20619	2442	10210	1125	898
		(31.2%)	(9.6%)	(25.5%)	(7.1%)	(58.4%)	(6.9%)	(28.9%)	(3.2%)	(2.5%)
	M	11356	3631	11896	3703	21663	2996	17986	1838	2418
		(32.1%)	(9.1%)	(29.8%)	(9.3%)	(54.3%)	(7.2%)	(45.1%)	(4.6%)	(6.1%)
75+	F	11933	2837	8056	5058	16806	4511	9477	1374	561
		(29.9%)	(8.2%)	(23.2%)	(14.6%)	(48.5%)	(13%)	(27.3%)	(4%)	(1.6%)
	M	23289	3548	11152	6610	18910	4954	16326	2257	1864
		(31%)	(8.9%)	(27.8%)	(16.5%)	(47.2%)	(12.4%)	(40.7%)	(5.6%)	(4.7%)

SABA: Short-Acting beta2-agonist, SAMA: Short- Acting Muscarinic Antagonist, LABA: Long- Acting beta2-agonist, LAMA: Long- Acting Muscarinic Antagonist, ICS: Inhaled corticosteroid, PDE-4: Phosphodiesterase inhibitor.

Note: The presented percentages refer to the patients of each age-group with specific gender who are receiving treatment with a specific group of active substances compared to patients of the same age-group and gender who don’t receive treatment.

63% patients in the age group of 35–54 years old were on LABA+ICS treatment, while 18,6% male patients and 28,5% female patients received LAMA. The percentage of LAMA users increased with age, while the use of LABA+ICS decreased. An increase with the age is also observed for patients receiving methylxanthine and PDE-4 medicines.

The correlation between COPD and the studied parameters (i.e. quantity of medicines, age, gender, the percentage of statutory participation and number of prescriptions according to therapeutic category) is presented in Table 4.

**Table 4:** Logarithmic regression results regarding the relationship between COPD and studied characteristics of the study population.

VARIABLE	RELATIVE RATIO	95% CONFIDENCE INTERVAL	WALD, p
NUMBER OF PACKAGES	1.6	(1.6, 16.5)	<0.001
STATUTORY PARTICIPATION	0.5	(0.49, 0.52)	<0.001
GENDER	1.25	(1.23, 1.27)	<0.001
AGE GROUP (35–54)	9.9	(9.3, 10.4)	<0.001
AGE GROUP (55–74)	18.4	(17.5, 19.3)	<0.001
AGE GROUP (75+)	23.6	(22.4, 25.1)	<0.001
ICS	1.1	(1.07, 1.12)	<0.001
SABA	1.56	(1.52, 1.61)	<0.001
LABA	4.3	(4.2, 4.4)	<0.001
SABA_SAMA	3.8	(3.7, 3.9)	<0.001
LABA_ICS	2.8	(2.7, 2.9)	<0.001
SAMA	2.1	(1.9, 2.13)	<0.001
LAMA	5.7	(5.5, 5.8)	<0.001
METHYLXANTHINE	2.1	(2.08, 2.3)	<0.001
PHOSPHODIESTERASE_4_INHIBITORS	3.7	(3.4, 4.03)	<0.001

All the studied characteristics have been found to be strongly associated with the presence of COPD, which could be also attributed to the large sample size in the present study.

More specifically, all studied parameters had a positive correlation with COPD, except the percentage of the statutory participation. Age increase was shown to be strongly correlated with the presence of COPD. Male presented higher possibility to develop COPD compared to female (Relative Ratio: 1.25 (95% CI, 1.23–1.27)). Regarding the active substance groups, patients receiving LAMA showed a stronger correlation with COPD.

The good-of-fitness check of the logarithmic model to the data showed adaptation in significant degree (p<0.001). The allocation ability of the model used has been tested and was sufficient.

Moreover, total pharmaceutical expenditure per active substance group-therapeutic category, gender and age group is presented in Table 5.

**Table 5:** Expenditure per therapeutic category, gender and age group.

CATEGORY	EXPENDITURE(Euros)
NUMBER OF PRESCRIPTIONS	22,308,459.81
PER THERAPEUTIC GROUP	
LABA &_ICS	9,963,742.10
ICS	4,671,540.51
LABA	4,186,949.65
LAMA	2,566,170.39
Phosphodiesterase_4_inhibitors	359,354.48
SABA & SAMA	258,808
SABA	197,926.56
SAMA	83,931.75
Methylxanthine	20,036.37
GENDER	
MALE	11,939,142.73
FEMALE	10,369,143.68
AGE GROUP (YEARS)	
35–54	2,475,990.07
55–74	9,934,568.32
75+	9,400,677.53

## Discussion

The present study presents highly reliable data on COPD prescribing patterns as the study population was comprised of more than 90% of the total insured population in Greece. The number of insurees with COPD receiving treatment for COPD (174,357), identified through a corresponding diagnosis on their prescription, compared to the total number of insurees (almost 9.9 million people) corresponds to a percentage of 1.75% during the study period. Given the reported prevalence rates of COPD at international level (7.6% with 95% CI 6–9.5%),[12] as well as the prevalence rates estimated in a smaller sample in Greece referring to adult (above 35 years old) smokers (total prevalence of COPD 8.4% with a variation from 6–10% in urban and rural areas, with urban areas presenting the largest incidence),[13] the present study, which identified 174,357 patients on treatment of 7,742,629 people covered by EOPYY during the study period, may show that a smaller percentage of patients with COPD actually receives treatment through the reimbursement organization.

Additionally, there seems to be a clear relationship between cost sharing and adherence and a study showed that an increasing patient share of medication costs was significantly associated with a decrease in adherence.[14]

With regards to physicians’ prescribing patterns, data in the present study showed that the majority of COPD patients were treated with a combination of LABA/ICS, followed by LAMA. This finding is different from a previous study conducted in Greece which found that LAMAs were the most prescribed drugs.[15] However, this could be attributed to the fact that GOLD 2011 recommendations have proposed the combination of LABA+ICS in patients for whom the same treatment will have been considered as overtreatment according to GOLD 2010 guideline. The use of LABA+ICS combination declines with age, while the use of LAMA increases. Differential diagnosis between asthma and COPD might be more prominent in younger population. A substantial proportion of patients clinically identified as having COPD in general practice do not have the condition according to spirometric criteria, with inaccurate diagnosis more common in patients with comorbidities. Policy and practice change is needed to support the use of spirometry in primary care.[16]

An additional finding of this study is the greater use of LAMA, among men which can possibly be attributed to more severe stage of COPD. In a recent study conducted in Greece according to GOLD classification 12.3% of patients were stage I, 39.6% were stage II, 34.9% stage III and 13.2% stage IV. 25.5% were assessed in patient group A, 13.2% in group B, 25.5% in group C and 35.8% in group C. (groups A,B,C,D are referring to grade of patients according to GOLD guidelines: A refers to low risk, low symptom burden, B refers to low risk, higher symptom burden, C refers to high risk, low symptom burden and D refers to high risk, higher symptom burden COPD patients) [17]

It should be stressed that LAMA are indicated only for COPD and not for asthma. These therapeutic categories include active substances, which are classified among the top 20 active substances in expenditure. Based on the distribution of therapeutic classes of medicines prescribed by physicians, it can be assumed that the majority of patients were classified in stages GOLD 3 or 4. Specifically, the LABA/ICS percentage is estimated at 48% (as ICS cannot be administered alone). The observed percentage is higher than expected as, according to a recent study referring to COPD outpatients receiving the combination LABA/ICS in Norway, the respective percentage was 39%,[18] while according to GOLD 2007 this is c. 18.6%, and in GOLD 2011 guidelines the LABA/ICS combination is suggested in moderate COPD.

The efficacy of LABA/ICS combination has also recently been evaluated in a Cochrane Collaboration Review[19] where it is mentioned that according to available data no therapeutic superiority has been demonstrated for the combination LABA/ICS compared to LABA alone for the prevention of exacerbations. On the other hand the increased risk of pneumonia associated with LABA/ICS is related to moderate quality evidence. In spite of a correlation of ICS use and increased risk of pneumonia,[20] a retrospective study revealed that COPD patients hospitalized for pneumonia who have used ICS in the past presented lower percentages of deaths and mechanical ventilation.[21] Thus, the use of ICS outweighs possible risks in some groups of COPD patients. An effort to classify COPD patients according to phenotypes will possibly guarantee the optimum use of LABA/ICS combination as maintenance therapy.[22] Finally, the WISDOM trial revealed that ICS do not reduce COPD exacerbations. This study revealed that although statistically significant difference in FEV1 was observed in the study group where the ICS was stepped down in 3 stages over 12 weeks from the combination LAMA+LABA it did not translate to changes in the exacerbation rate.[23]

A recent network meta-analysis which compared long-acting inhaled therapy (LABA, LAMA, ICS) for COPD outlined that quality of life and lung function were improved most on combination inhalers (LABA and ICS) and least on ICS alone at 6 and at 12 months. Overall LAMA and LABA inhalers had similar effects, particularly at 12 months. The network has demonstrated the benefit of ICS when added to LABA for these outcomes in participants who largely had an FEV1 that was less than 50% predicted, but the additional expense of combination inhalers and any potential for increased adverse events require consideration.[24],[25]

Recently, new standard combinations of LAMA/LABA have entered the market of COPD assuring better compliance and ease of use (once a day administration regimens). However, possible greater cardiovascular risks should be taken into account.

Moreover, the estimated market share of COPD prescriptions dispended by private pharmacies during the study period corresponds to 2% of the total expenditure in private pharmacies. The highest expenditure is due to prescriptions of LABA/ICS combination, followed by LAMA, while the target population in aspects of pharmaceutical expenditure for COPD is above 55 years old. A study conducted in the UK indicated that for ICS-tolerant patients the cost-effectiveness frontier suggested LAMA as initial treatment. Where patients continue to exacerbate and additional therapy is required, LAMA + LABA/ICS can be a cost-effective option, followed by LAMA + LABA/ICS + roflumilast [26]. The slight lower cost for COPD female patients compared to males could be attributed in the difference in COPD percentage. Additionally, according to recent data presented in a Hellenic Congress on 2014 the total expenditure for ATC4 category R03AK (Adrenergics in combination with corticosteroids) is 28,9 million € for the first semester of the year for both COPD and asthma indication. Of course, for EOPYY as a reimbursement fund covering nowadays above 90% of the Hellenic population would be a cost-effective method to proceed through negotiations, extra rebates, price volume and performance based agreements in the case of medicines for the treatment of COPD. Until now there is not a tender procedure in place, but the recent establishment of a Negotiation Committee could trigger the procedure.

In Greece, generic substitution is allowed in the pharmacy level and the pharmacist is obliged by law to indicate the generic with the lowest price. However, this cannot change the initial choice of the physician regarding the active substance.

The major strength of the present study is the large sample size, while major limitations refer to a) difficulties in data extraction, as the information was extracted from a big data database not specifically structured for research purposes, and b) to the fact that the reporting of diagnosis based on ICD-10 coding was not mandatory for physicians in 2012.

It should be noted that the unification of individual SSFs into one, i.e. EOPYY, went together with the evolution and implementation of electronic prescribing in the country. In particular, in January 2012 e-prescriptions corresponded to 44% of total prescriptions while in December 2012 they corresponded to 85% of the total number of prescriptions issued in the country. The observed equalization of percentage of COPD between males and females could be attributed to the increase of female smokers as confirmed in previous studies.[11]

Moreover, it has been previously observed that when the statutory co-payment level differs between diseases (in case of Greece 0%, 10%, 25% depending on the disease) patients may be issued a prescription with a false diagnosis in order to take advantage of reduced co-payment. This was the case for asthma and COPD in the first trimester of 2012. Specifically, during the first months of 2012 there was a difference in patients’ contribution for asthma (25%) and COPD (10%), which was later equalized by a legislative change in October 2012. Therefore, there could be an overestimation of COPD cases in the study. This finding triggers the initiative to develop and codify automated filters/boundaries in the electronic prescription system based on age (as depicted by the social security number) and diagnosis (based on ICD-10 classification) for COPD.

This study also highlights the potential role of big data in public, health policy making and shows the capacity to extract epidemiologically and well-characterized, representative populations. As big data analysis is a hypothesis generating machine we need to move from the validity to the utility of evidence generated.[27]

## Conclusion

In conclusion, this study provides real-world evidence on the number of patients with COPD receiving treatment in Greece, estimates its correlation with age, gender, number of packages and active substance group prescribed, shows possible gender differences in treatment response and disease severity, indicates the necessity of appropriate COPD classification in primary care, and illustrates the role of electronic prescribing as a tool of ensuring appropriate prescribing and, therefore, of improving health outcomes.

## References

[1] World Health Organization (WHO). Programs-Chronic Respiratory Diseases. Accessed March 7, 2014. Available from: http://www.who.int/respiratory/copd/en/

[2] National Clinical Guideline Centre. Chronic obstructive pulmonary disease: management of chronic obstructive pulmonary disease in adults in primary and secondary care. London: National Clinical Guideline Centre 6 2010 Available from: http://guidance.nice.org.uk/CG101/Guidance/pdf/English

[3] Diaz-Guzman E, Mannino DM. Epidemiology and Prevalence of Chronic Obstructive Pulmonary Disease. Clin Chest Med. 2014 3;35(1):7–16. 10.1016/j.ccm.2013.10.002 24507833

[4] Murray CJL, Lopez AD, editors. The global burden of disease: a comprehensive assessment of mortality and disability from diseases, injuries and risk factors in 1990 and projected to 2020 The Global Burden of Disease and Injury Series: v.1. Cambridge, MA: Harvard University Press, 1996.

[5] Punekar YS, Shukla A, Müllerova H. COPD management costs according to the frequency of COPD exacerbations in UK primary care. Int J Chron Obstruct Pulmon Dis. 2014;9:65–73. 10.2147/COPD.S54417 24426781 PMC3890403

[6] Geitona M, Hatzikou M, Steiropoulos P, Alexopoulos EC, Bouros D. The cost of COPD exacerbations: a university hospital—based study in Greece. Respir Med. 2011 3;105(3):402–9. 10.1016/j.rmed.2010.09.020 20970310

[7] Halbert RJ, Natoli JL, Gano A, Badamgarav E, Buist AS, Mannino DM. Global burden of COPD: systematic review and meta-analysis. Eur Respir J. 2006 9;28(3):523–32. 16611654 10.1183/09031936.06.00124605

[8] European Respiratory Society. European Lung White Book: Huddersfield, European Respiratory Society Journals, Ltd; 2003.

[9] National Clinical Guideline Centre. Chronic obstructive pulmonary disease: management of chronic obstructive pulmonary disease in adults in primary and secondary care. London: National Clinical Guideline Centre 6 2010 Available from: http://guidance.nice.org.uk/CG101/Guidance/pdf/English.

[10] Tzanakis N, Anagnostopoulou U, Filaditaki V, Christaki P, Siafakas N; COPD group of the Hellenic Thoracic Society. Prevalence of COPD in Greece. Chest. 2004 3;125(3):892–900. 15006947 10.1378/chest.125.3.892

[11] Papaioannou AI, Bania E, Alexopoulos EC, Mitsiki E, Malli F, Gourgoulianis KI. Sex discrepancies in COPD patients and burden of the disease in females: a nationwide study in Greece (GreekObstructive Lung Disease Epidemiology and health ecoNomics: GOLDEN study). Int J Chron Obstruct Pulmon Dis. 2014 2 17;9:203–13. 10.2147/COPD.S52500 24600217 PMC3933352

[12] Raherison C, Girodet PO. Epidemiology of COPD. Eur Respir Rev 2009; 18 (114): 213–221. 10.1183/09059180.00003609 20956146

[13] Tzanakis N, Anagnostopoulou U, Filaditaki V, Christaki P, Siafakas N; COPD group of the Hellenic Thoracic Society. Prevalence of COPD in Greece. Chest. 2004 3;125(3):892–900. 15006947 10.1378/chest.125.3.892

[14] Eaddy MT, Cook CL, O'Day K, Burch SP, Cantrell CR. How patient cost-sharing trends affect adherence and outcomes: a literature review. P T. 2012 1;37(1):45–55. 22346336 PMC3278192

[15] Papala M., Kerenidi N., Gourgoulianis KI. Everyday clinical practice and its relationship to 2010 and 2011 GOLD guideline recommendations for the management of COPD. Prim Care Respir J. 2013 9; 22(3):362–4. 10.4104/pcrj.2013.00073 23989678 PMC6442835

[16] Zwar NA, Marks GB, Hermiz O, Middleton S, Comino EJ, Hasan I et al Predictors of accuracy of diagnosis of chronic obstructive pulmonary disease in general practice. Med J Aust. 2011 8 15;195(4):168–71. 21843115 10.5694/j.1326-5377.2011.tb03271.x

[17] Stafyla E, Kerenidi T, Gourgoulianis KI. Chronic obstructive pulmonary disease exacerbation frequency and severity. Int J Chron Obstruct Pulmon Dis. 2013; 8:533–5. 10.2147/COPD.S53318 24235823 PMC3825673

[18] Drivenes E, Ostrem A. Predictors of ICS/LABA prescribing in COPD patients: a study from general practice. BMC Fam Pract. 2014; 15: 42 10.1186/1471-2296-15-42 24597538 PMC3983887

[19] Nannini LJ, Lasserson TJ. Combined corticosteroid and long-acting beta2-agonist in one inhaler versus long-acting beta2-agonists for chronic obstructive pulmonary disease. Cochrane Database of Systematic Reviews 2012, Issue 9. Art. No.: CD006829. 10.1002/14651858.CD006829 17943918

[20] Zab Mosenifar. Chronic Obstructive Pulmonary Disease Treatment & Management. Medscape. Last update 09.05.2014. Available from: http://emedicine.medscape.com/article/297664-treatment#aw2aab6b6b3.

[21] Chen D, Restrepo MI, Fine MJ, Pugh MJ, Anzueto A, Metersky ML et al Observational study of inhaled corticosteroids on outcomes for COPD patients with pneumonia. Am J Respir Crit Care Med. 8 1 2011;184(3):312–6. 10.1164/rccm.201012-2070OC 21512168 PMC3175534

[22] Miravitlles M, Calle M, Soler-Cataluna JJ. Clinical phenotypes of COPD: identification, definition and implications for guidelines. Arch Bronconeumol. 2012;15:86–98. 10.1016/j.arbres.2011.10.007 22196477

[23] Magnussen H, Disse B, Rodriguez-Roisin R, Kirsten A, Watz H, Tetzlaff K et al; WISDOM Investigators. Withdrawal of inhaled glucocorticoids and exacerbations of COPD. N Engl J Med. 2014 10 2;371(14):1285–94. 10.1056/NEJMoa1407154 25196117

[24] Kew KM, Dias S, Cates CJ. Long-acting inhaled therapy (beta-agonists, anticholinergics and steroids) for COPD: a network meta-analysis. Cochrane Database Syst Rev. 2014 3 26;3:CD010844 10.1002/14651858.CD010844.pub2 24671923 PMC10879916

[25] Kew KM, Seniukovich A. Inhaled steroids and risk of pneumonia for chronic obstructive pulmonary disease. Cochrane Database Syst Rev. 2014 3 10;3:CD010115 10.1002/14651858.CD010115.pub2 24615270 PMC8966042

[26] Hertel N, Kotchie RW, Samyshkin Y, Radford M, Humphreys S, Jameson K. Cost-effectiveness of available treatment options for patients suffering from severe COPD in the UK: a fully incremental analysis. Int J Chron Obstruct Pulmon Dis. 2012;7:183–99. 10.2147/COPD.S29820 22500119 PMC3325000

[27] Khoury MJ, Ioannidis JP.Medicine. Big data meets public health. Science 2014 11 28; 346 (6213): 1054–5. 10.1126/science.aaa2709 25430753 PMC4684636

